# Real-time thermal coronary angiography in coronary artery bypass grafting

**DOI:** 10.1093/icvts/ivaf078

**Published:** 2025-03-28

**Authors:** Rakan I Nazer, Ali M Albarrati

**Affiliations:** Department of Cardiac Science, King Fahad Cardiac Center, College of Medicine, King Saud University, Riyadh, Kingdom of Saudi Arabia; Department of Rehabilitation Science, College of Applied Medical Science, King Saud University, Riyadh, Kingdom of Saudi Arabia

**Keywords:** Thermal imaging, Coronary artery bypass grafting, Quality assurance

## Abstract

Intraoperative graft interrogation is crucial for ensuring graft patency and optimizing surgical outcomes in coronary artery bypass grafting (CABG). Various intraoperative imaging techniques aid in assessing graft function, allowing for immediate correction of technical issues and reducing postoperative complications. A 38-year-old male smoker presented with acute coronary syndrome, characterized by central chest pain radiating to the left shoulder and ST-segment depression on electrocardiogram. Elevated troponin confirmed the diagnosis, and coronary angiography revealed significant 3-vessel disease. The patient underwent urgent CABG using bilateral internal mammary arteries as a Y-graft to the left anterior descending artery and the first diagonal branch. Additional grafts to the posterior descending artery and first obtuse marginal branch were performed using saphenous vein segments. Intraoperative graft patency was assessed using a real-time thermal imaging camera, providing non-invasive visualization of myocardial blood flow without contrast agents or disruption of the surgical workflow. This unique technique allowed immediate confirmation of graft functionality, ensuring quality assurance during CABG. Thermal imaging potentially represents a state-of-the-art tool for improving graft quality and surgical outcomes, offering a simple, effective alternative to conventional intraoperative imaging methods.

## CASE PRESENTATION

A 38-year-old male smoker presented to the emergency department with central chest pain radiating to the left shoulder, which awoke him from sleep. On evaluation, his vital signs were stable, but his electrocardiogram showed ST-segment depression in the lateral leads. High-sensitivity troponin I was elevated at 20 ng/l, which was consistent with acute coronary syndrome. Coronary angiography revealed significant 3-vessel disease, and echocardiography demonstrated preserved left ventricular function. The patient was subsequently scheduled for urgent coronary artery bypass grafting (CABG). Post-surgery, the patient had an uneventful postoperative recovery. He was discharged home on day 6 after surgery. He continues to be stable at 24 months after surgery.

## OPERATIVE DETAILS

The patient underwent on-pump CABG with cross-clamp using bilateral internal mammary arteries configured as a Y-graft to revascularize the left anterior descending artery and the first diagonal branch (D1). Additional bypasses to the posterior descending artery and the first obtuse marginal branch (OM1) were performed using saphenous vein grafts harvested from the right leg.

## INTRAOPERATIVE CORONARY IMAGING

**Figure 1:**
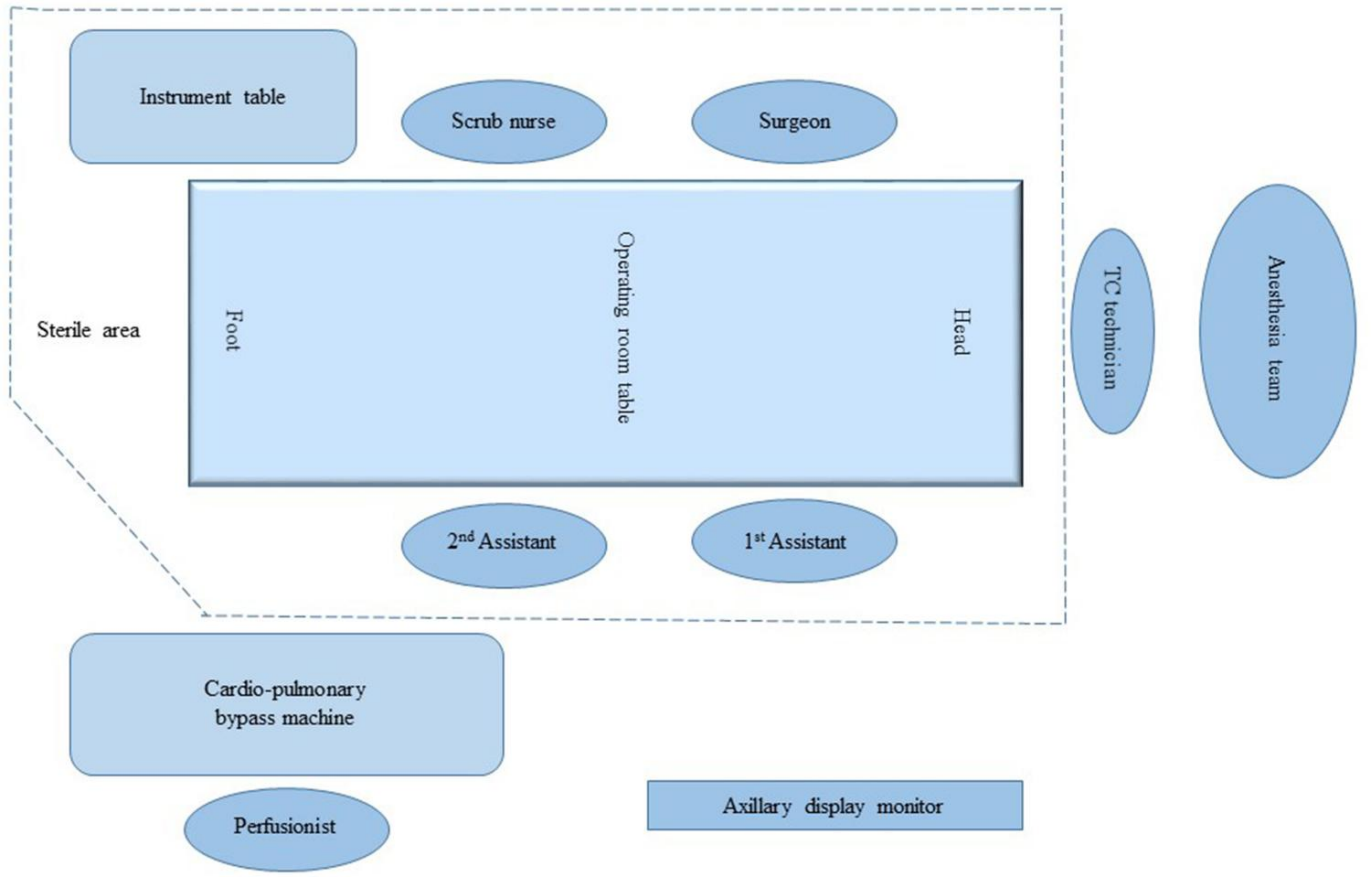
A schematic illustration of the cardiac operating room setup for real-time coronary thermal angiography in coronary bypass surgery. TC: thermal camera.

**Figure 2:**
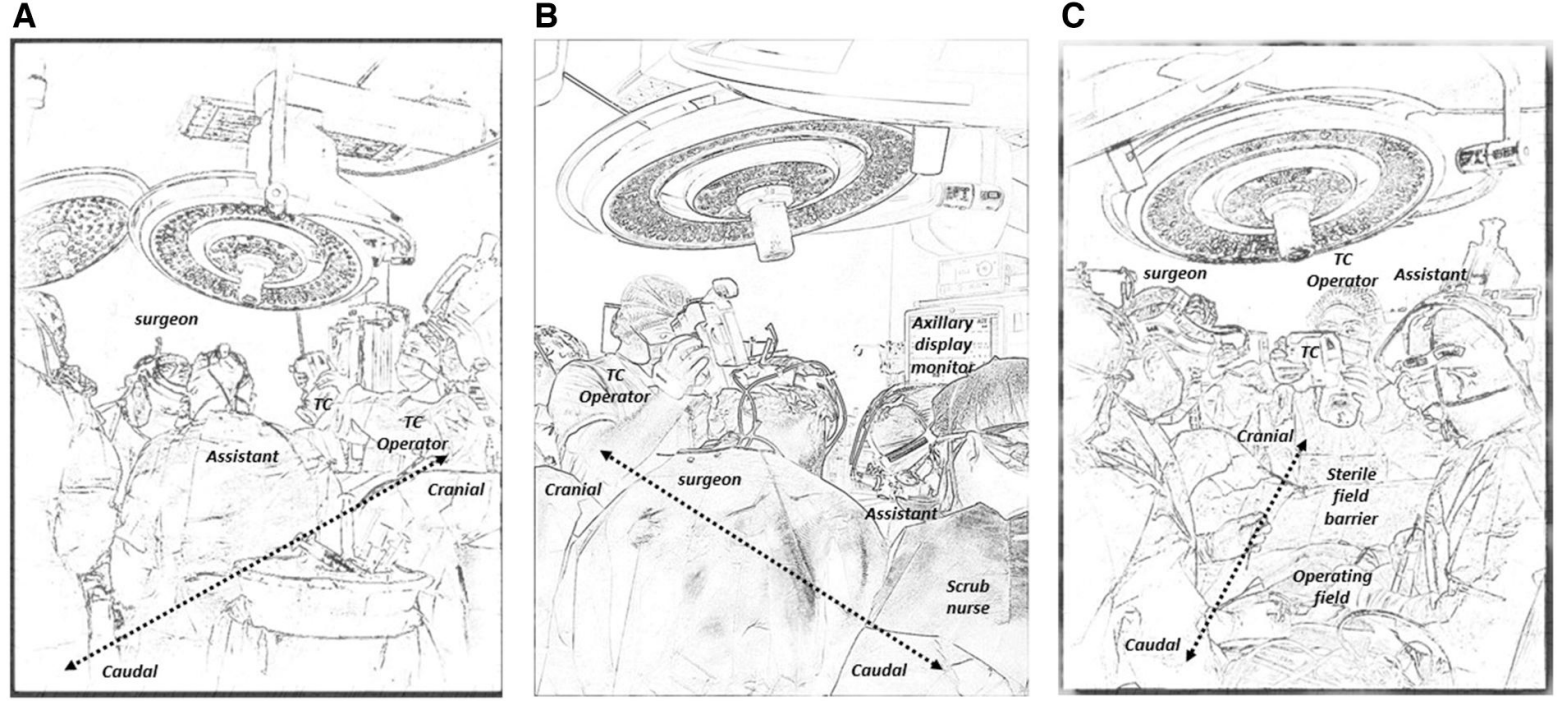
An illustrating sketch or the cardiac operating room showing ongoing real-time coronary thermal angiography in coronary bypass surgery. (**A**) View of from the left side of the operating table showcasing the position of the operating surgeon, assistant and the TC operator. (**B**) View from the right side of the operating table. (**C**) View from the foot of the operating room table. TC: thermal camera.

To ensure intraoperative graft patency, we utilized a real-time thermal imaging camera (FLIR T540, Teledyne FLIR LLC, USA) for real-time visualization of blood flow through the grafts. The Thermal Camera was provided by the manufacturer as a part of a pilot study to assess the potential application of this modality in coronary surgery. The camera was handled by a dedicated trained technical assistant, who held it behind the sterile drapes overlooking the surgical field to record detailed thermal images of the anterior surface of the heart. The images were on display on a digital monitor inside the operating theatre where the surgical team can view the thermal display for reference (Figs 1 and 2).

The systemic temperature on cardiopulmonary bypass was kept normothermic. Topical cooling was applied on the surface of the heart after induction of the cardiac arrest with cold potassium-blood cardiopleagia. Flow in the vein grafts was checked by manually injecting warm saline after each distal anastomosis. Flow in the bilateral internal mammary Y-graft was checked after the release of the micro-vascular clamp (Video 1).

## DISCUSSION

Intraoperative graft assessment methods, such as transit-time flowmetry (TTFM) and fluorescence imaging, have demonstrated utility in detecting technical failures and ensuring graft functionality. However, these techniques have limitations. For instance, TTFM relies on haemodynamic parameters that can be influenced by systemic factors, while fluorescence imaging requires contrast agents and may prolong operative time [1].

Thermal imaging addresses some of these challenges by leveraging temperature differences to visualize blood flow in real time. Studies have shown its effectiveness in identifying graft patency and anastomotic issues during off-pump and on-pump CABG [2, 3]. In this case, the use of thermal imaging allowed for real-time intraoperative detection of graft flow. This has the potential for detecting technical abnormalities graft function and flow, enabling immediate revisions and reducing the risk of postoperative complications.

While thermal imaging has been utilized for over 2 decades, its adoption remains limited. This could be due to cost and logistical challenges. Unlike TTFM, which is simple and requires no additional trained personnel, thermal angiography necessitates specialized equipment and trained staff, increasing costs. In this study, the system was provided and operated by the manufacturer, but broader adoption may reduce costs over time. Its potential to improve graft quality assurance and reduce complications could enhance cost-effectiveness, warranting further research into its clinical and economic impact. Unlike the other modalities for intraoperative coronary imaging, there are no long-term studies verifying the impact of this modality on outcomes. Thermal imaging enables real-time detection of graft-related issues, allowing immediate correction and improving intraoperative quality assurance. While cost remains a challenge, its potential to enhance outcomes and reduce complications warrants further evaluation [4]. Advances in camera technology and imaging resolution, as demonstrated in this case, underscores its potential for broader clinical application. Although we believe all grafts should be subjected to quality interrogation before leaving the operating room, the importance of graft quality becomes more evident when using multiple arterial grafting, sequential anastomosis and small distal targets. Future research should focus on direct comparisons with other modalities, such as TTFM and fluorescence imaging, to establish standardized protocols and assess long-term outcomes.

**Conflict of interest:** none declared.

## Data Availability

All data related to this report are available up on request.
